# Supplementary material for: Daily exposure to stressors, daily perceived severity of stress, and mortality risk among US adults
Source: PLoS One. 2024 May 15;19(5):e0303266. doi: 10.1371/journal.pone.0303266 (PMC11095670; doi:10.1371/journal.pone.0303266)
Supplement: S1 Text — (PDF) [file pone.0303266.s001.pdf]

# S1 TEXT. MIDUS MAIN SURVEY AND NSDE

The MIDUS data are publicly available at https://www.icpsr.umich.edu/web/ICPSR/series/203 (see links below to the various data files used in this analysis).

## Main Survey

### *Original Cohort*

At Wave 1 (https://doi.org/10.3886/ICPSR02760.v19), MIDUS targeted non-institutionalized, English-speaking adults aged 25-74[1] in the contiguous United States [1]. National random digit dialing with oversampling of older people and men was used to select the main sample (*N*=3487) and a sample of twin pairs (*N*=1914). The study also included a random subsample of siblings of individuals in the main sample (*N*=950) and oversamples from five metropolitan areas in the U.S. (*N*=757). The response rate for the phone interview (fielded January 1995–September 1996) ranged from 60% for the twin subsample to 70% for the main sample. Among those who completed the phone interview (*N*=7108), 6325 (89%) also completed a mail-in self-administered questionnaire (SAQ) [2].

At Wave 2 (https://doi.org/10.3886/ICPSR04652.v8), the MIDUS cohort was re-contacted: 4963 (75% of 6628 survivors) completed a follow-up telephone interview (fielded January 2004–August 2005) and 4041 (81% of those who completed the phone interview; 61% of survivors) completed the SAQ.

At Wave 3 (https://doi.org/10.3886/ICPSR36346.v7), the cohort was contacted again; 3294 (56% of 5929 survivors) completed the telephone interview (fielded May 2013–April 2014) and 2732 (83% of those who completed the phone interview; 49% of survivors) completed the SAQ [2].

### *Milwaukee Cohort*

A new oversample of African Americans in Milwaukee was recruited at Wave 2 (https://doi.org/10.3886/ICPSR22840.v6) [3]. Computer-assisted personal interviewing (CAPI) was used to conduct an in-person interview (fielded April-October 2005) with 592 respondents aged 35-83 (71% response rate); 416 (70% of those who completed the CAPI) also completed the SAQ.

At Wave 3 (https://doi.org/10.3886/ICPSR37120.v4), 389 (79% of 492 survivors) were re-interviewed via CAPI (fielded June 2016-January 2017) and 327 (84% of those who completed the CAPI; 66% of survivors) also completed the SAQ.

## National Study of Daily Experiences (NSDE)

The NSDE was a sub-study of MIDUS that collected a daily diary of stressful experiences. A representative subset of respondents who completed the main survey at each wave were selected to participate in the NSDE. The vast majority of NSDE participants also completed the SAQ of the main survey (99.9% at Wave 1, 96.4% at Wave 2, 96.7% at Wave 3). At each NSDE wave, an end-of-day telephone interview was conducted on eight consecutive days. The data

[1] Although MIDUS targeted Americans aged 25-74, the final sample included a few respondents aged 20 (*N*=2), 23 (*N*=1), 24 (*N*=12), or 75 (*N*=4) at the time of the Wave 1 phone interview.

from Waves 1-3 of the NSDE are publicly available at https://doi.org/10.3886/ICPSR03725.v6; https://doi.org/10.3886/ICPSR26841.v2; and https://doi.org/10.3886/ICPSR38529.v1.

## REFERENCES

1. Brim OG, Baltes PB, Bumpass LL, Cleary PD, Featherman DL, Hazzard WR, et al. National Survey of Midlife Development in the United States (MIDUS 1), 1995-1996: Description of MIDUS Samples. Inter-university Consortium for Political and Social Research [distributor], Version 19. 2020 [cited 28 Jul 2021]. Available: https://doi.org/10.3886/ICPSR02760.v19

2. Ryff C, Almeida DM, Ayanian JS, Carr DS, Cleary PD, Coe C, et al. Midlife Development in the United States (MIDUS 2), 2004-2006. ICPSR04652, Version 7. Ann Arbor, MI: Inter-university Consortium for Political and Social Research [distributor]. Ann Arbor, MI: Inter-university Consortium for Political and Social Research [distributor]; 2017. doi:10.3886/ICPSR04652.v7

3. Ryff C, Almeida D, Ayanian J, Carr DS, Cleary PD, Coe C, et al. Midlife in the United States (MIDUS 2): Milwaukee African American Sample, 2005-2006 (ICPSR 22840), Version 6. Inter-University Consortium for Political and Social Research; 2022. doi:https://doi.org/10.3886/ICPSR22840.v6
