# Supplementary material for: Daily exposure to stressors, daily perceived severity of stress, and mortality risk among US adults
Source: PLoS One. 2024 May 15;19(5):e0303266. doi: 10.1371/journal.pone.0303266 (PMC11095670; doi:10.1371/journal.pone.0303266)
Supplement: S2 Text — (PDF) [file pone.0303266.s002.pdf]

S2 TEXT. MORTALITY FOLLOW-UP

The National Death Index (NDI) search was based on final death data for 1995-2021. Respondents who were still alive at Wave 2 or 3, but did not participate in the NSDE for that wave were censored at the end of the NSDE fieldwork for that wave (April 2009 for Wave 2, September 2019 for Wave 3). There was one respondent **(M2ID**=18721) recorded as having died in 2014, but s/he participated in Wave 3 of the NSDE in 2018. We recoded this respondent as alive, but censored this individual at Wave 3 of NSDE because we have no information regarding vital status after that.

The most recent mortality file for MIDUS (https://doi.org/10.3886/ICPSR37237.v4) also includes deaths based on an early release data file for 2022. Although we have data for deaths occurring as late as December 2022, mortality after 12/31/2021 is likely to be incomplete. The previous NDI match was based on final death data through 2019 and an early release file for 2020, which was estimated to include approximately 95% of recorded US deaths in 2020. Now that we have the final death data for 2020, we were able to confirm that the sensitivity was, in fact, only 86%. Among all the recorded deaths in 2020 among the MIDUS sample (based on the final death data), 14% of them were missing from the early release file for 2020. Thus, the level of completion of the early release file for 2022 remains unknown. We hope the sensitivity will be better than in was in 2020 because that was a very challenging year for everyone, including the agencies tracking and coding deaths. Nonetheless, we suspect that the early release file is far from complete. At this point, any analyses of mortality after December 31, 2021 would need to be interpreted cautiously.

The ICD-9 and ICD-10 codes that define the cause-specific mortality groups are listed below:

**Cause-Specific Group**	**ICD-9 Codes**	**ICD-10 Codes**	**N of Deaths**
Extrinsic mortality	001-139, 162, 180, 260-269, 280-281, 283.1-283.2, 291, 303, 304, 320-322, 390-398, 460-519, 571, 590, 595, 597-599, 601, 630-676, 725, 730, E800-E999	A00-A09, A16-A44, A48-A99, B00-B09, B15-B99,C33-C34, C53, D50-D53, D59, D86.9, E40-E64, F10-F16, F19, G00, G02, G03, G04.2, G14, H32, I00-I09, I32, I39, J00-J06, J09-J18, J20-J22, J30-J47, J60-J98, K20, K62, K70, K73-K74, K75.4, K90.8, L44.4, L94.6, M02.3, M35.2, M35.3, M46, M66.0, M83.9, M86, M90.8, N10-N12, N13.6, N13.9, N15.1, N15.9, N16.0, N21.9, N28.8, N30, N34.1, N35, N36, N37, N39, N40.1, N41, N51, N99.1, O00-O08, R11.1, R31, U01-U03, U07.1, V01-Y98, Z33.2	141
Intrinsic mortality (i.e., residual)	All other ICD-9 codes	All other ICD-10 codes	410

Our definition of extrinsic mortality differs somewhat from Masters et al. [1]. For example, we added COVID-19 (ICD-10: U07.1), which did not exist when that paper was published. For some reason, Masters et al. excluded some codes for external causes (e.g., ICD-9: E979; ICD-10: V02-V04, V09.0, V09.2, V12-V14, V19.0-V19.2, V19.4-V19.6, V20-V79, V80.3-V80.5, V81.0, V81.1, V82.0-V82.1, V87.0-V87.8, V88.0-V88.8, V89.0, V89.2, Y89.0, Y90-Y98). We included all deaths from external causes (ICD-9: E800-E999; ICD-10: V01-Y98) as extrinsic mortality.

REFERENCES

1. Masters RK. Explaining recent mortality trends among younger and middle-aged white Americans. Int J Epidemiol. 2018;47: 81–88. doi:10.1093/ije/dyx127
