# Supplementary material for: Daily exposure to stressors, daily perceived severity of stress, and mortality risk among US adults
Source: PLoS One. 2024 May 15;19(5):e0303266. doi: 10.1371/journal.pone.0303266 (PMC11095670; doi:10.1371/journal.pone.0303266)
Supplement: S3 Text — (PDF) [file pone.0303266.s003.pdf]

# S3 TEXT. MEASURE OF SOCIOECONOMIC STATUS

Relative SES was based on education of the respondent (and spouse/partner, if applicable), current or most recent occupation of the respondent (and spouse/partner, if applicable), annual household income, and current net wealth of the respondent and spouse. Education and occupation were asked in the main interview, whereas income and wealth came from the SAQ. Education is based on degree completion with 12 response categories ranging from less than 8th grade to completion of a professional degree (e.g., PhD, MD, JD, etc.). We recoded occupation into four groups:

1=Farming/Construction/Maintenance/Production/Transportation/Military;
2=Service/Sales/Administrative/Office;
3=Management/Business/Financial;
4=Professional.

Annual household income includes all sources of income for the respondent, spouse/partner, and all other family members living in the household. Total net wealth is reported for the respondent and spouse/partner combined. (See next section for more details regarding income and assets.) We standardized all six items and calculated the mean across relevant items (e.g., six items if married/partnered and both respondent and spouse/partner have ever been employed; three items if not married/partnered and respondent has never been employed; Cronbach's $\alpha$=0.72 at M1, 0.75 at M2, 0.74 at M3). Finally, within each survey wave, we converted the composite score to a percentile rank (1-100). Then, we rescaled it to range from 0 (bottom percentile) to 1 (top percentile) such that a one-unit change denotes the difference between the top and bottom percentile of SES.

## Income and Wealth

At each wave, income from each source (i.e., wages/salary, social security, government assistance, and all other sources such as pensions, investments, child support, or alimony) was reported in categories, which we recoded to the mid-point of the range within each category.

### *Top-Coding of Income*

At Waves 1 and 2, income from each source was top-coded at $200,000 (except government assistance, which was top-coded at $50,000; for the Milwaukee subsample added at Wave 2, they also top-coded at lower values: $100,000 for wages/salary; $50,000 for pension, $40,000 for social security, $30,000 for government assistance). At Wave 3, all income sources were top-coded at $300,000 (for both the core and the Milwaukee subsample). Top-coded income from one or more sources comprised 1.1% of the analysis sample for Wave 1, 2.1% at Wave 2, and 1.6% at Wave 3.

Top-coded values are recoded to the harmonic mean of a Pareto distribution. As suggested by von Hippel et al. [1], we computed the harmonic mean of a Pareto distribution with $\alpha$ equal to the maximum of one or $[\ln(n_{B-1} + n_B) - \ln(n_B)] \div [\ln(l_B) - \ln(l_{B-1})]$, where $n_B$ is the number of cases in the top category; $n_{B-1}$ is the number of cases in the penultimate category; $l_B$ is the lower bound of the top category; and $l_{B-1}$ is the lower bound of the penultimate category. Restricting alpha to a minimum of one ensures that the value of the top category is no greater than twice the lower bound of that category. We then summed across all sources to compute total income. We were unable to make an equivalence adjustment based on household size and composition because MIDUS did not collect that information at Wave 1.

### *Bottom- and Top-Coding of Wealth*

Net wealth was also reported in categories at Wave 1 and coded to the mid-point of each range. There were separate categories for “less than $0 [loss]” and “$0 [None]”, both of which we coded to zero. At Waves 2 and 3, the respondent was asked to report the dollar amount of net wealth, but values below -$300,000 were bottom-coded, which we retained as -$300,000.

At all three waves, wealth was top-coded at $1,000,000 (2.8% of the analysis sample for Wave 1, 9.2% at Wave 2, 18.6% at Wave 3), which we recoded to the harmonic mean of a Pareto distribution as described above for income.

To adjust for inflation, we converted income and assets to 1995 dollars using the Consumer Price Index (CPI) provided by the Bureau of Labor Statistics (https://data.bls.gov/cgi-bin/cpicalc.pl). For each respondent, we determined the multiplier for income/assets based on the year in which s/he completed the phone interview and the CPI multiplier for the median month for MIDUS interviews conducted during that year (using April 1995 as the reference, which was the median month among interviews completed in 1995). Thus, the multipliers for each survey year for the core sample (based on the median month for interviews in that year) were: 1995 (April)=1.0; 1996 (July)=0.97; 2004 (May)=0.80; 2005 (March)=0.79; 2013 (July)=0.65; 2014 (March)=0.64. For the Milwaukee subsample, the multipliers were 2005 (June)=0.78; 2016 (Sept)=0.63; 2017 (Jan)=0.63.

## REFERENCES

1. von Hippel PT, Scarpino SV, Holas I. Robust estimation of inequality from binned incomes. Sociological Methodology. 2016;46: 212–251. doi:10.1177/0081175015599807
