# Supplementary material for: Daily exposure to stressors, daily perceived severity of stress, and mortality risk among US adults
Source: PLoS One. 2024 May 15;19(5):e0303266. doi: 10.1371/journal.pone.0303266 (PMC11095670; doi:10.1371/journal.pone.0303266)
Supplement: S4 Text — (PDF) [file pone.0303266.s004.pdf]

S4 TEXT. MEASURES OF HEALTH STATUS

At each wave, the respondent was asked about various chronic conditions and physical limitations. Based on data from the main interview, we included dichotomous measures indicating whether the respondent ever had 1) cancer; and 2) heart trouble (suspected or confirmed by a doctor). Using data collected in the SAQ, we included binary variables indicating whether the respondent reported the following conditions during the 12 months prior to the interview: 3) stroke; 4) diabetes; and 5) lung problems (i.e., asthma, bronchitis, emphysema, tuberculosis, or other lung problems).

Physical limitations were based on the following questions asked in the SAQ, "How much does your health limit you in doing each of the following? Lifting or carrying groceries; climbing several flights of stairs; bending, kneeling, or stooping; walking more than a mile; walking several blocks; walking one block; vigorous activity (e.g., running, lifting heavy objects); moderate activity (e.g., bowling, vacuuming)." The response categories for each of the eight physical tasks were coded on a four-point scale (0=not at all, 1=a little, 2=some, 3=a lot). Based on Long and Pavalko [1], we constructed an index by summing the eight items (potential range 0–24), adding a constant (0.5), and taking the logarithm of the result, which allows for relative rather than absolute effects. Then, we standardized the resulting scores based on the pooled distribution across all three waves.

REFERENCES

1. Long JS, Pavalko E. Comparing alternative measures of functional limitation. MedCare. 2004;42: 19–27. doi:10.1097/01.mlr.0000102293.37107.c5
