# Supplementary material for: Daily exposure to stressors, daily perceived severity of stress, and mortality risk among US adults
Source: PLoS One. 2024 May 15;19(5):e0303266. doi: 10.1371/journal.pone.0303266 (PMC11095670; doi:10.1371/journal.pone.0303266)
Supplement: S5 Text — (PDF) [file pone.0303266.s005.pdf]

S5 TEXT. MULTIPLE IMPUTATION

Among the 4,756 observations included in the analysis, the predictors with the highest percentage of missing data were household income (30%),[1] wealth (17%), and spouse's occupation (7%); missing data was less than 5% for all other variables. We used the Stata user-written "ice" command to perform multiple imputation. For the multiple imputation process, we used information for all the analysis variables as well as auxiliary measures of employment status of the respondent and spouse/partner; wages/salary income of respondent and of the spouse/partner; and self-assessed health status.

For continuous variables, departures from normality may result in implausible imputations when using the default draw method. To reduce the skewness, we applied an inverse hyperbolic sine (IHS) transformation[2] for measures of income and wealth. Several other variables had a skewed distribution (i.e., exposure to and reported severity of the seven stressors). To ensure that imputed values were within the range of observed values, we used prediction matching for all those variables and several others for which imputation generated out of range values (i.e., birth cohort, educational attainment, measures of income and wealth, index of physical limitations).

We used an ordered logit model for imputation of ordinal variables (e.g., occupation category, self-assessed health status). We performed five imputations and then used the "mi estimate" prefix command to re-estimate the model for each imputation and combine the five sets of estimates using Rubin's rules [4].

REFERENCES

1. Killewald A, Pfeffer FT, Schachner JN. Wealth inequality and accumulation. Annu Rev Sociol. 2017;43: 379–404. doi:10.1146/annurev-soc-060116-053331

2. Friedline T, Masa RD, Chowa GAN. Transforming wealth: Using the inverse hyperbolic sine (IHS) and splines to predict youth's math achievement. Social Science Research. 2015;49: 264–287. doi:10.1016/j.ssresearch.2014.08.018

3. Pence KM. The Role of Wealth Transformations: An Application to Estimating the Effect of Tax Incentives on Saving. The BE Journal of Economic Analysis & Policy. 2006;5: 1–26.

[1] As noted in S3 Text, household income is based on the summation across a variety of income sources (i.e., wages/salary, social security, pension, government assistance, other income sources). A smaller percentage of the sample was missing data for wages/salary of the respondent and the spouse/partner, but there were higher levels of missing data for other income sources, especially for other family members in the household. The SAQ instructed the respondent to enter $0 for any income sources that were not applicable, but it is likely that some respondents simply left those items blank if there was no income from that source (e.g., no other family members in the household or those other family members did not contribute any income). When we sum across all household income sources, 9% of the NSDE respondents at Wave 1 were missing data from at least one income source but that percentage rose to 40% among NSDE respondent at Wave 2 and Wave 3.

[2] Both income and wealth were positively skewed, but we cannot apply a log-transformation to zero or negative values. Killewald et al.[1] advise against recoding negative values to a small positive values in order to apply a log-transformation. Instead, they suggest using an inverse hyperbolic sine (IHS) transformation, which can incorporate zero and negative values. The IHS function is approximately linear near zero and similar to the logarithm for large values [2,3].

4. Royston P, Carlin JB, White IR. Multiple imputation of missing values: new features for mim. Stata Journal. 2009;9: 252–264.
