# Supplementary material for: Daily exposure to stressors, daily perceived severity of stress, and mortality risk among US adults
Source: PLoS One. 2024 May 15;19(5):e0303266. doi: 10.1371/journal.pone.0303266 (PMC11095670; doi:10.1371/journal.pone.0303266)
Supplement: S1 File — (ZIP) [file pone.0303266.s006.zip › S6_file/Related/MyNotes-NSDE_M1.docx]

NSDE, Wave 1

At M1, 1499 Rs participated in the NSDE (fielded March 1996-March 1997); a random subset of 1843 Rs who completed the main survey (from RDD and Twin samples) were selected for NSDE (81% response rate). See <https://midus-study.github.io/public-documentation/M1P2/Documentation/M1_P2_UserGuide_20211213.pdf> for documentation of NSDE at M1. [Note: 99.9% (N=1497) of Wave 1 NSDE participants also completed the SAQ of the main survey.]

Each night for 8 consecutive nights (via telephone interview), R was asked questions about:

- Time use, giving, volunteering (Section A)
- Physical Health (Section B)
- Non-specific Psychological distress (Section C, A2DC1-A2DC12 at M1)
- Work productivity and cutbacks (Section D)
- Stressful experiences (Section F, A2DF1-A2DF7, see pp. 52-69 of the User Guide PDF)
  - Severity of each perceived stressor (A2DF1C-A2DF7C)
  - How much it disrupted your daily routine (A2DF*D), financial situation (A2DF*E), how you feel about yourself (A2DF*F), how other people feel about you (A2DF*G), your physical health/safety (A2DF*H), health/wellbeing of someone you care about (A2DF*I), and plans for the future (A2DF*J).
- Review of the week on the Final Day (Sections H, I, & J)

See pp. 31-75 of the User’s Guide for the codebook (don’t show us the instrument?).

# Constructed Measures (pp. 114-130 of the User Guide PDF)

- Total # of stressors [**A2DTOTST**]: Counts how many of the 7 stressors R reports experiencing each day. [SEEMS TO HAVE CODING ERRORS]
- Any stressors [**A2DANYST**]: Dummy indicating whether R reported any of the 7 stressors each day. [SEEMS TO HAVE CODING ERRORS]
- Mean severity of stressors (expert coders) [**A2DSEVAV**]: mean severity (coded from 1=low to 4=extreme) across all 7 items. [CANNOT CHECK CODING BECAUSE SOURCE VARS ARE NOT INCLUDED IN THE DATAFILE]
- Mean severity of stressors (as rated by R) [**A2DXFCAV**]: mean severity (coded from 1=not at all to 4=very) across all 7 items. [DOES NOT APPEAR TO BE COMPUTED CORRECTLY]
- Note: Corr b/w **A2DSEVAV** & **A2DXFCAV** is ???.

# stress Measures

Asked 7 open-ended Qs about daily events (see p. 11 of the User Guide PDF): 1) argument/disagreement with someone; 2) avoided argument; 3) something happened at work/school that most people would consider stressful; 4) something happened at home that most people would consider stressful; 5) experienced discrimination; 6) something happened to a close friend or relative that turned out to be stressful for you; 7) anything else happened that most people would consider stressful.

Some prior studies (e.g., Lockwood et al. 2022, <https://www.ncbi.nlm.nih.gov/pmc/articles/PMC9528775/>) have used a simple dummy for whether R experienced any of the 7 stressors each day or counted the number of stressors reported across the 8-day period (Chiang et al. 2018), but it seems to me that it would be better to use more of the information. They asked Rs about the severity of each stressor. Why not use that information? There is a constructed variable for mean stressor severity as rated by the R [**A2DXFCAV**]. There is also a constructed variable for mean stressor severity as rated by experts [**A2DSEVAV**]—what does that mean???. Why not multiply the average # of perceived stressors per day by the average severity of the stressors to obtain a measure of cumulative exposure/intensity of perceived stress?

In the model predicting mortality, we could include the average # of stressors per day (measure of perceived stress exposure) and the cumulative perceived intensity of the stressor (i.e., # of stressors per day x average severity per stressor).

## Prior Studies

- Lockwood et al. (2022, <https://www.ncbi.nlm.nih.gov/pmc/articles/PMC9528775/>) constructed a dummy variable indicating whether R reported any of the 7 types of stressors on each day during the NSDE (Rs reported no stressors on 62% of days and only 10% reported multiple stressors on any given day). According to Table 1, the mean # of stressor days was 2.8 (SD=2.0).
- Almeida et al. (Almeida et al. 2023) call it “stressor exposure”, but it seems more like perceived stress to me (whether or not an event is “stressful” is subject to the R’s subjective evaluation, which could be influenced by other factors; it is not an objective measure of exposure to stressors).
- (Chiang et al. 2018): Counted the number of stressors reported across the 8-day period.

I have never used any of the data from the National Study of Daily Experiences (NSDE), but I looked at some of David Almeida’s previous publications to see how they are using the measures. For example, Lockwood et al. (2022) also constructs the following measures:

1. **Daily negative affect** was computed as the mean across 14 items (coded 0-4) from the nonspecific psychological distress scale (Kessler et al. 2002, *Psychol Med*) and the indexes for positive and negative affect (Watson et al. 1988) on each day during the NSDE (day level α=0.91). According to Table 1, the mean daily negative affect was 0.2 (SD=0.3).

Other studies (e.g., <https://midus.wisc.edu/findings/pdfs/1742.pdf>, <https://midus.wisc.edu/findings/pdfs/1819.pdf>) have used negative affect reactivity (i.e., the extent to which reported levels of negative affect are higher stressor days relative to non-stressor days).

# Dataset

Data: ~\Box\MIDUS\M1\P2 (NSDE)\ICPSR_03725-V6\DS0001\03725-001-Data.dta

Do-file: ~\Box\MIDUS\M1\P2 (NSDE)\NSDE_M1.do

N=10,488 observations (one for each day with up to 8 observations per R)

- [**Nstress**] I recomputed the number of stressors reported by R per day [because **A2DTOTST** appears to have coding errors; for example, **A2DTOTST** was not calculated for 173 RS (e.g., M2ID=10503), but it is not clear why because there are valid responses for all 7 Qs; in a couple other cases (M2ID=10424, A2DDAY=7; M2ID=10454, A2DDAY=4), A2DTOTST was coded as 0 but R refused all 7 Qs; in 84 other cases (e.g., M2ID=10433, A2DDAY=2), **A2DTOTST** is inconsistent with the responses to **A2DF1**-**A2DF7**]. We coded this variable as missing for 0.7% of the observations because R refused 1+ of the 7 Qs upon which is it based. Among the observations with valid data, 62% report no stressor, 27% report 1 stressor, 8% report 2 stressors, and 3% report 3+ stressors.
- [**Anystress**] Similarly, I recomputed the dummy indicating whether R reported any stressor each day [because **A2DANYST** appears to have similar coding errors].
- [**StressSev**] I recomputed the cumulative severity of stressors (as rated by the R) [again, because **A2DXFCAV** does not appear to be computed correctly]. First, I recoded each of the individual items (**A2DF1C A2DF2C A2DF3B A2DF4B A2DF5C A2DF6C A2DF7B**) to range from 0 (Not at all) to 3 (very/a lot). If R did not report experiencing that stressor on that day, I recoded it to 0. Then, I summed across the 7 items. We coded this variable as missing for 78 (0.7%) observations because R refused 1+ of the 7 Qs. Theoretically, the final score ranges from 0 to 21 (but the max score is 15—that is, “somewhat” across the 7 Qs or “very” for 5 stressors). Most observations (64%) had a score of 0 (mostly because no stressor was reported that day); 22% scored 1-2 (e.g., “not very/a little” or “some/somewhat” on 1 stressor); and 14% scored 3+ (e.g., “very/a lot” on 1 stressor).

## Observations missing data for all 7 stressors

There were 24 observations for whom there was no valid data for any of the 7 stressors. Most of them seem to have valid data from some of the other vars in the NSDE. Thus, we assume that they just refused the stress Qs for some reason.

If we were to drop these observations, it would leave 10,464 observations for 1,499 Rs.

The dataset (~\Box\MIDUS\M1\P2 (NSDE)\NSDE_M1.dta) includes 1 rec for each observed day for each R (N=10,464 obs):

Contains data from C:\Users\dglei\Box\MIDUS\M1\P2 (NSDE)\NSDE_M1.dta

obs: 10,488 M1, NSDE: Stress vars for each R on each of up to 8 days

(N=10,488 obs)

vars: 20 21 Nov 2023 11:35

------------------------------------------------------------------------------------------------------------

storage display value

variable name type format label variable label

------------------------------------------------------------------------------------------------------------

M2ID long %5.0f MIDUS 2 ID number

DAY int %1.0f Day of interview

NSDE_WAVE str2 %9s

NSDEym float %tm Yr/Mo of NSDE Wave 1

F1r int %9.0g RECODE of A2DF1 (Did you have an argument or disagreement?)

F2r int %9.0g RECODE of A2DF2 (Did you avoid a disagreement?)

F3r int %9.0g RECODE of A2DF3 (Anything stressful happen at work or school?)

F4r int %9.0g RECODE of A2DF4 (Did anything stressful happen at home?)

F5r int %9.0g RECODE of A2DF5 (Did you experience discrimination-race,sex,

or age?)

F6r int %9.0g RECODE of A2DF6 (Stressful event happen to close friend or

relative that affecte

F7r int %9.0g RECODE of A2DF7 (Did anything else stressful happen to you?)

Nstress float %9.0g Total # of stressors reported per day (out of 7)

F1Cr int %10.0g Severity RECODE of A2DF1C (Argument/disagreement-How stressful was this

for you?)

F2Cr int %10.0g Severity RECODE of A2DF2C (Avoid disagreement-How stressful was this

for you?)

F3Br int %10.0g Severity RECODE of A2DF3B (Stress at work-How stressful was this for

you?)

F4Br int %10.0g Severity RECODE of A2DF4B (Stress at home-How stressful was event?)

F5Cr int %10.0g Severity RECODE of A2DF5C (Discrimination-How stressful was this

incident?)

F6Cr int %10.0g Severity RECODE of A2DF6C (Stress to friend-How stressful was this for

you?)

F7Br int %10.0g Severity RECODE of A2DF7B (Other stress-How stressful was this for

you?)

StressSev float %9.0g Sum of severity scores across 7 stressors

## Compute Average Across Observed Days for Each R

- For each R, we computed:
  - [**stress_days**] # of observed days for **Nstress**
    - Only 49% of Rs have valid data for **Nstress** on all 8 days. Most (93%) have data for at least 5 days. Fewer than 2% have valid data for less than 2 days.
  - [**Nstress**] Per day average # of stressors (across observed days for each R).
    - 11% of Rs reported no stressors on any observed day; 82% reported an average of less than 1 stressor per day; 2% of Rs reported an average of 2+ stressors per day (max was 3.86).
  - [**Sev_days**] # of observed days for **StressSev**
    - Distribution very similar to **stress_days**. Only 48% of Rs have valid data for **StressSev** on all 8 days. Most (93%) have data for at least 5 days. Fewer than 2% have valid data for less than 2 days.
  - [**StressSev**] Per day average perceived severity of stress (across all observed days for each R).
    - 14% of Rs reported 0 severity; 59% scored less than 1 (e.g., average of less than “not very” on 1 stressor); and 5% score 3+ (e.g., average of very on one stressor across all observed days; max=7.1).

The final dataset (~\Box\MIDUS\M1\P2 (NSDE)\NSDE_R_M1.dta) includes 1 record for each of 1499 Rs:

Contains data from C:\Users\dglei\Box\MIDUS\M1\P2 (NSDE)\NSDE_R_M1.dta

obs: 1,499 M1, NSDE: Stressor measures for each R (N=1,499)

vars: 22 21 Nov 2023 11:35

------------------------------------------------------------------------------------------------------------

storage display value

variable name type format label variable label

------------------------------------------------------------------------------------------------------------

M2ID long %5.0f MIDUS 2 ID number

NSDE_WAVE str2 %9s

NSDEym_M1 float %tm Yr/Mo of NSDE Wave 1

stress_days_M1 long %9.0g # of observed days for Nstress

Sev_days_M1 long %9.0g # of observed days for StressSev

F1r_M1 float %9.0g (mean) F1r

F2r_M1 float %9.0g (mean) F2r

F3r_M1 float %9.0g (mean) F3r

F4r_M1 float %9.0g (mean) F4r

F5r_M1 float %9.0g (mean) F5r

F6r_M1 float %9.0g (mean) F6r

F7r_M1 float %9.0g (mean) F7r

F1Cr_M1 float %10.0g (mean) F1Cr

F2Cr_M1 float %10.0g (mean) F2Cr

F3Br_M1 float %10.0g (mean) F3Br

F4Br_M1 float %10.0g (mean) F4Br

F5Cr_M1 float %10.0g (mean) F5Cr

F6Cr_M1 float %10.0g (mean) F6Cr

F7Br_M1 float %10.0g (mean) F7Br

Nstress_M1 float %9.0g Cumulative # stressors (sum of per day mean for each stressor)

StressSev_M1 float %9.0g Cumulative severity (sum of per day mean for each stressor)

NSDE_M1 float %9.0g

------------------------------------------------------------------------------------------------------------

# References

Almeida, David M., Jonathan Rush, Jacqueline Mogle, Jennifer R. Piazza, Eric Cerino, and Susan T. Charles. 2023. “Longitudinal Change in Daily Stress across 20 Years of Adulthood: Results from the National Study of Daily Experiences.” *Developmental Psychology* 59(3):515–23. doi: 10.1037/dev0001469.

Chiang, Jessica J., Nicholas A. Turiano, Daniel K. Mroczek, and Gregory E. Miller. 2018. “Affective Reactivity to Daily Stress and 20-Year Mortality Risk in Adults with Chronic Illness: Findings from the National Study of Daily Experiences.” *Health Psychology* 37(2):170–78. doi: 10.1037/hea0000567.
