# Supplementary material for: Daily exposure to stressors, daily perceived severity of stress, and mortality risk among US adults
Source: PLoS One. 2024 May 15;19(5):e0303266. doi: 10.1371/journal.pone.0303266 (PMC11095670; doi:10.1371/journal.pone.0303266)
Supplement: S1 File — (ZIP) [file pone.0303266.s006.zip › S6_file/Related/MyNotes-NSDE_M2.docx]

NSDE, Wave 2

At M2, 2022 Rs participated in the NSDE (fielded April 2004-April 2009, but most interviews were between July 2004 and Oct 2008) contributing 16,176 observations (up to 8 days per R). Wave 2 of NSDE targeted a representative subset of Rs who completed the main survey (including RDD, twins, siblings, & MKE subsamples); they do not provide the response rate (see Study Description). [Note: They say they only selected Rs who completed both the main interview and the SAQ, but I find that only 96.4% (N=1950) of Wave 2 NSDE participants completed the SAQ.]

Each night for 8 consecutive nights (via telephone interview), R was asked questions about:

- Time use, giving, volunteering (Section A)
- Physical Health, cigarette & alcohol use (Section B)
- Non-specific Psychological distress **and positive affect** (Section C)
- Work productivity and cutbacks (Section D)
- Stressful experiences (Section F)
- Review of the week on the Final Day (Sections H, I, J & K)
- Discrimination (Section S)

See “26841-…tele_interview.pdf” for the instrument.

“26841-…description.pdf” says that approx. 69% of Rs completed interviews on all 8 days.

## Salivary Cortisol (New at M2)

Starting at M2, they also collected salivary cortisol (4 times x 4 days)—see <https://www.icpsr.umich.edu/web/NACDA/studies/26841/versions/V2/datasets/1/files/1245415/downloadDoc/doc?path=/pcms/studies/0/2/6/8/26841/V2/files/1245415>.

# Constructed Measures

(see “26841-…-scales.pdf”)

- Total # of stressors [**B2DN_STR**, p. 4]: Counts how many of the 7 stressors R reports experiencing each day. [I don’t use their constructed variable because they treated “DK/refused/missing” as if it meant there was no stressor.]
- Any stressors [**B2DA_STR**, p. 6]: Dummy indicating whether R reported any of the 7 stressors each day.
- Mean severity of stressors (as rated by R) [**B2DSSEVS**, p. 8]: mean severity (coded from 0=not at all to 3=very) across all 7 items. [ignored DK/refused missing and computed severity only if R reported a stressor]

# Dataset

~\Box\MIDUS\M2\P2 (NSDE)\ICPSR_26841-V2\DS0001\26841-001-Data.dta

N=16,176 observations (2,022 Rs with one observation for each of up to 8 days)

NOTE: N=1264 of those observations apparently did not complete the phone interview (i.e., **D2DMISS**=1). We dropped those observations from the dataset (leaving 14,912 observations for 2,022 Rs).

- [**Nstress**] I recomputed the number of stressors reported by R per day [because **B2DN_STR** treats DK/refused/missing as no stressor]. We coded this variable as missing for 0.6% of the observations because R refused 1+ of the 7 Qs upon which it is based. Among the observations with valid data, 61% report no stressor, 29% report 1 stressor, 8% report 2 stressors, and 2% report 3+ stressors.
- [**Anystress**] Similarly, I recomputed the dummy indicating whether R reported any stressor each day [because **B2DA_STR** treats DK/refused/missing as no stressor].
- [**StressSev**] I recomputed average severity (as rated by the R) [because **B2DSSEVS** ignored DK/refused missing and computed severity only if R reported a stressor]. First, I recoded each of the individual items (**B2DF1C B2DF2C B2DF3B B2DF4B B2DF5C B2DF6C B2DF7B**) to range from 0 (Not at all) to 3 (very/a lot). If R did not report experiencing that stressor on that day, I recoded it to 0. Then, I summed across the 7 items. We coded this variable as missing for 0.6% observations because R refused 1+ of the 7 Qs. Theoretically, the final score ranges from 0 to 3 (but the max score is 13—that is, approx. “somewhat” across the 7 Qs). Most observations (64%) had a score of 0 (mostly because no stressor was reported that day); 22% scored 1-2 (e.g., “not very/a little” or “some/somewhat” on 1 stressor); and 14% scored 3+ (e.g., “very/a lot” on 1 stressor).

## Observations missing data for all 7 stressors

There were another 14 observations for whom there was no valid data for any of the 7 stressors Most of the other vars also appear to be missing for these observations.

If we were to drop these observations, it would leave 14,898 observations for 2,022 Rs.

The dataset (~\Box\MIDUS\M2\P2 (NSDE)\NSDE_M2.dta) includes 1 rec for each observed day for each R (N=14,898 obs):

Contains data from C:\Users\dglei\Box\MIDUS\M2\P2 (NSDE)\NSDE_M2.dta

obs: 14,912 M2, NSDE: Stress vars for each R on each of up to 8 days

(N=14,912 obs)

vars: 20 21 Nov 2023 11:38

------------------------------------------------------------------------------------------------------------

storage display value

variable name type format label variable label

------------------------------------------------------------------------------------------------------------

M2ID long %5.0f MIDUS 2 ID number

DAY int %5.0f B2DDAY Interview day

NSDE_WAVE str2 %9s

NSDEym float %tm Yr/Mo of NSDE Wave 1

F1r int %9.0g RECODE of B2DF1 (Did you have an argument/disagreement?)

F2r int %9.0g RECODE of B2DF2 (Did you avoid a disagreement?)

F3r int %9.0g RECODE of B2DF3 (Did anything happen at work/school?)

F4r int %9.0g RECODE of B2DF4 (Did anything happen at home?)

F5r int %9.0g RECODE of B2DF5 (Did any discrimination happened to you?)

F6r int %9.0g RECODE of B2DF6 (Did anything happen to friend stres you?)

F7r int %9.0g RECODE of B2DF7 (Did anything else happen to you?)

Nstress float %9.0g Total # of stressors reported (out of 7)

F1Cr int %10.0g Severity RECODE of B2DF1C (How stressful was this for you?)

F2Cr int %10.0g Severity RECODE of B2DF2C (How stressful was this for you?)

F3Br int %10.0g Severity RECODE of B2DF3B (How stressful was this for you?)

F4Br int %10.0g Severity RECODE of B2DF4B (How stressful was this for you?)

F5Cr int %10.0g Severity RECODE of B2DF5C (How stressful was this for you?)

F6Cr int %10.0g Severity RECODE of B2DF6C (How stressful was this for you?)

F7Br int %10.0g Severity RECODE of B2DF7B (How stressful was this for you?)

StressSev float %9.0g Sum of severity scores across 7 stressors

-------------------------------------------------------------------------------------------------------------

## Compute Average Across Observed Days for Each R

- For each R, we computed:
  - [**stress_days**] # of observed days for **Nstress**
    - 66% of Rs have valid data for **Nstress** on all 8 days.
  - [**Nstress**] Per day average # of stressors (across observed days for each R).
    - 10% of Rs reported no stressors on any observed day; 84% reported an average of less than 1 stressor per day; 2% of Rs reported an average of 2+ stressors per day (max was 5).
  - [**Sev_days**] # of observed days for **StressSev**
    - Distribution very similar to **stress_days**. 66% of Rs have valid data for **StressSev** on all 8 days.
  - [**StressSev**] Per day average perceived severity of stress (across all observed days for each R).
    - 14% of Rs reported 0 severity (mostly because they reported no stressors on any day); 60% scored less than 1 (e.g., average of less than “not very” on 1 stressor); and 5% score 3+ (e.g., average of very on one stressor across all observed days; max=9).

The final dataset (~\Box\MIDUS\M2\P2 (NSDE)\NSDE_R_M2.dta) includes 1 record for each of 2022 Rs:

Contains data from C:\Users\dglei\Box\MIDUS\M2\P2 (NSDE)\NSDE_R_M2.dta

obs: 2,022 M2, NSDE: Stressor measures for each R (N=2,022)

vars: 22 21 Nov 2023 11:38

------------------------------------------------------------------------------------------------------------

storage display value

variable name type format label variable label

------------------------------------------------------------------------------------------------------------

M2ID long %5.0f MIDUS 2 ID number

NSDE_WAVE str2 %9s

NSDEym_M2 float %tm Yr/Mo of NSDE Wave 1

stress_days_M2 long %9.0g # of observed days for Nstress

Sev_days_M2 long %9.0g # of observed days for StressSev

F1r_M2 float %9.0g (mean) F1r

F2r_M2 float %9.0g (mean) F2r

F3r_M2 float %9.0g (mean) F3r

F4r_M2 float %9.0g (mean) F4r

F5r_M2 float %9.0g (mean) F5r

F6r_M2 float %9.0g (mean) F6r

F7r_M2 float %9.0g (mean) F7r

F1Cr_M2 float %10.0g (mean) F1Cr

F2Cr_M2 float %10.0g (mean) F2Cr

F3Br_M2 float %10.0g (mean) F3Br

F4Br_M2 float %10.0g (mean) F4Br

F5Cr_M2 float %10.0g (mean) F5Cr

F6Cr_M2 float %10.0g (mean) F6Cr

F7Br_M2 float %10.0g (mean) F7Br

Nstress_M2 float %9.0g Cumulative # stressors (sum of per day mean for each stressor)

StressSev_M2 float %9.0g Cumulative severity (sum of per day mean for each stressor)

NSDE_M2 float %9.0g

------------------------------------------------------------------------------------------------------------
