# Supplementary material for: Daily exposure to stressors, daily perceived severity of stress, and mortality risk among US adults
Source: PLoS One. 2024 May 15;19(5):e0303266. doi: 10.1371/journal.pone.0303266 (PMC11095670; doi:10.1371/journal.pone.0303266)
Supplement: S1 File — (ZIP) [file pone.0303266.s006.zip › S6_file/Related/MyNotes-NSDE_M3.docx]

NSDE, Wave 3

At M3, 1236 Rs participated in the NSDE (fielded March 2017-Sept 2019) contributing 9,301 observations (up to 8 days per R). Wave 3 of NSDE targeted a random subset of respondents who completed the main survey; they do not provide the response rate (see Study Description). [Note: 96.7% of Wave 3 NSDE participants also completed the SAQ of the main survey.]

Each night for 8 consecutive nights (via telephone interview), R was asked questions about:

- Time use, giving, volunteering (Section A)
- Physical Health, cigarette & alcohol use (Section B)
- Non-specific Psychological distress **and positive affect** (Section C)
- Work productivity and cutbacks (Section D)
- Stressful experiences (Section F)
- Review of the week on the Final Day (Sections H, I, J & K)
- Discrimination (Section S)

See “M3_P2_...Instrument...pdf” for the instrument.

“M3_P2-…description...pdf” says that 76% of Rs completed interviews on all 8 days.

# Constructed Measures

(see “M3_P3…Scales...pdf”)

- Total # of stressors [**C2DN_STR**, p. 4]: Counts how many of the 7 stressors R reports experiencing each day.
- Any stressors [**C2DA_STR**, p. 6]: Dummy indicating whether R reported any of the 7 stressors each day.
- Mean severity of stressors (as rated by R) [**C2DSSEVS**, p. 8]: mean severity (coded from 0=not at all to 3=very) across all 7 items. [ignored DK/refused missing and APPEARS TO HAVE CODING ERRORS]

# Dataset

~\Box\MIDUS\M3\P3 (NSDE)\M3_P2_DailyDiary_N1236_20220323.dta (converted from SPSS)

N=9,301 observations (1,236 Rs with one observation for each of up to 8 days)

- [**Nstress**] Number of stressors reported by R per day (matches **C2DN_STR** exactly). We coded this variable as missing for 568 (6.1%) observations because R refused 1+ of the 7 Qs upon which is it based. Among the observations with valid data, 62% report no stressor, 29% report 1 stressor, 7% report 2 stressors, and <2% report 3+ stressors.
- [**Anystress**] Similarly, I recomputed the dummy indicating whether R reported any stressor each day [because **C2DA_STR** treats DK/refused/missing as no stressor].
- [**StressSev**] I recomputed average severity (as rated by the R) [because **C2DSSEVS** ignored DK/refused missing and appears to be inconsistent with the original variables—it is not clear how they come up with the values they got!]. The individual items (**C2DF1C C2DF2C C2DF3B C2DF4B C2DF5C C2DF6C C2DF7B**) were already coded to range from 0 (Not at all) to 3 (very/a lot). If R did not report experiencing that stressor on that day, I recoded it to 0. Then, I summed across the 7 items. We coded this variable as missing for 571 (6.1%) observations because R refused 1+ of the 7 Qs. Theoretically, the final score ranges from 0 to 21 (but the max score is 15—that is, approx. “somewhat” across the 7 Qs). Most observations (64%) had a score of 0 (mostly because no stressor was reported that day); 24% scored 1-2 (e.g., “not very/a little” or “some/somewhat” on 1 stressor); and 12% scored 3+ (e.g., “very/a lot” on 1 stressor).

## Observations missing data for all 7 stressors

Why are there so many observations (N=499) with no valid data for any of the 7 stressors?

- Most of them are also missing most other variables as well including the Qs at the beginning about sleep (C2DWAKE* C2DAW1*) & other vars asked before the stress Qs (e.g., C2DA1H, C2DB1A, C2DC1, C2DD1).
- The only vars for which they seem to have lots of valid cases are follow-up questions that I think should have been coded N/A because the screener Q is missing. For example, 482 Rs were missing for time spent giving unpaid assistance (C2DA10), but they were coded “NO” on C2DA10A1 (gave assistance to spouse).

. tab C2DA10 C2DA10A1 if miss_number==14, missing

Did you spend | Who you gave unpaid

time giving | assist? Spouse

unpaid assist? | NO . | Total

----------------+----------------------+----------

YES | 1 0 | 1

NO | 9 0 | 9

. | 482 7 | 489

----------------+----------------------+----------

Total | 492 7 | 499

- Maybe these Rs missed the phone interview (but there is no indicator variable [**C2DMISS**], like there was for Wave 2)?

If we were to drop these observations, it would leave 8,802 observations for 1,174 Rs. [There were 62 Rs who were missing all stress vars for all days (60 had only 1 day of data, 2 had 3 days of data).]

The dataset (~\Box\MIDUS\M3\P2 (NSDE)\NSDE_M3.dta) includes 1 rec for each observed day for each R (N=8,802 obs):

Contains data from C:\Users\dglei\Box\MIDUS\M3\P2 (NSDE)\NSDE_M3.dta

obs: 9,301 M3, NSDE: Stress vars for each R on each of up to 8 days

(N=9,301 obs)

vars: 20 21 Nov 2023 11:39

------------------------------------------------------------------------------------------------------------

storage display value

variable name type format label variable label

------------------------------------------------------------------------------------------------------------

M2ID int %5.0f MIDUS 2 ID number

DAY byte %8.0f labels3 Interview day

NSDE_WAVE str2 %9s

NSDEym float %tm Yr/Mo of NSDE Wave 1

F1r byte %9.0g RECODE of C2DF1 (Did you have an argument/disagreement?)

F2r byte %9.0g RECODE of C2DF2 (Did you avoid a disagreement?)

F3r byte %9.0g RECODE of C2DF3 (Did anything happen at work/school?)

F4r byte %9.0g RECODE of C2DF4 (Did anything happen at home?)

F5r byte %9.0g RECODE of C2DF5 (Did any discrimination happened to you?)

F6r byte %9.0g RECODE of C2DF6 (Did anything happen to friend stress you?)

F7r byte %9.0g RECODE of C2DF7 (Did anything else happen to you?)

Nstress float %9.0g Total # of stressors reported (out of 7)

F1Cr byte %10.0g Severity RECODE of C2DF1C (How stressful was this for you?)

F2Cr byte %10.0g Severity RECODE of C2DF2C (How stressful was this for you?)

F3Br byte %10.0g Severity RECODE of C2DF3B (How stressful was this for you?)

F4Br byte %10.0g Severity RECODE of C2DF4B (How stressful was this for you?)

F5Cr byte %10.0g Severity RECODE of C2DF5C (How stressful was this for you?)

F6Cr byte %10.0g Severity RECODE of C2DF6C (How stressful was this for you?)

F7Br byte %10.0g Severity RECODE of C2DF7B (How stressful was this for you?)

StressSev float %9.0g Sum of severity scores across 7 stressors

------------------------------------------------------------------------------------------------------------

## Compute Average Across Observed Days for Each R

- For each R, we computed:
  - [**stress_days**] # of observed days for **Nstress**
    - Only 72% of Rs have valid data for **Nstress** on all 8 days.
  - [**Nstress**] Per day average # of stressors (across observed days for each R).
    - 15% of Rs reported no stressors on any observed day; 84% reported an average of less than 1 stressor per day; <2% of Rs reported an average of 2+ stressors per day (max was 5).
  - [**Sev_days**] # of observed days for **StressSev**
    - Distribution very similar to **stress_days**. 71% of Rs have valid data for **StressSev** on all 8 days.
  - [**StressSev**] Per day average perceived severity of stress (across all observed days for each R).
    - 17% of Rs reported 0 severity; 62% scored less than 1 (e.g., average of less than “not very” on 1 stressor); and <4 score 3+ (e.g., average of very on one stressor across all observed days; max=14).

The final dataset (~\Box\MIDUS\M3\P2 (NSDE)\NSDE_R_M3.dta) includes 1 record for each of 1174 Rs:

Contains data from C:\Users\dglei\Box\MIDUS\M3\P2 (NSDE)\NSDE_R_M3.dta

obs: 1,236 M3, NSDE: Stressor measures for each R (N=1,236)

vars: 22 21 Nov 2023 11:39

------------------------------------------------------------------------------------------------------------

storage display value

variable name type format label variable label

------------------------------------------------------------------------------------------------------------

M2ID int %5.0f MIDUS 2 ID number

NSDE_WAVE str2 %9s

NSDEym_M3 float %tm Yr/Mo of NSDE Wave 1

stress_days_M3 long %9.0g # of observed days for Nstress

Sev_days_M3 long %9.0g # of observed days for StressSev

F1r_M3 float %9.0g (mean) F1r

F2r_M3 float %9.0g (mean) F2r

F3r_M3 float %9.0g (mean) F3r

F4r_M3 float %9.0g (mean) F4r

F5r_M3 float %9.0g (mean) F5r

F6r_M3 float %9.0g (mean) F6r

F7r_M3 float %9.0g (mean) F7r

F1Cr_M3 float %10.0g (mean) F1Cr

F2Cr_M3 float %10.0g (mean) F2Cr

F3Br_M3 float %10.0g (mean) F3Br

F4Br_M3 float %10.0g (mean) F4Br

F5Cr_M3 float %10.0g (mean) F5Cr

F6Cr_M3 float %10.0g (mean) F6Cr

F7Br_M3 float %10.0g (mean) F7Br

Nstress_M3 float %9.0g Cumulative # stressors (sum of per day mean for each stressor)

StressSev_M3 float %9.0g Cumulative severity (sum of per day mean for each stressor)

NSDE_M3 float %9.0g

------------------------------------------------------------------------------------------------------------
