# Supplementary material for: Daily exposure to stressors, daily perceived severity of stress, and mortality risk among US adults
Source: PLoS One. 2024 May 15;19(5):e0303266. doi: 10.1371/journal.pone.0303266 (PMC11095670; doi:10.1371/journal.pone.0303266)
Supplement: Attachment — Submitted filename: Response to Reviewers-2024-03-28.pdf [file pone.0303266.s007.pdf]

REVIEWER #1

1. This paper is well done.
2. Just thinking broadly, could the findings be different among women, white or Hispanic non-white populations? The introductory part could incorporate more information by comparing gender, race among other demographic variables. Older adult population is very diverse and doing a comparative analysis could reveal insights for future policy, research and practice implications.

Response: We agree that doing a comparative analysis could reveal insights for future. Unfortunately, we have limited statistical power to examine whether the associations between stress exposure/perceived severity and mortality differ by sex or by race/ethnicity.

One prior study (Nielsen et al. 2008, which we cited in the Introduction) reported an association among men but not women; they also noted that the sex interaction with stress was significant at *p*~0.02. In the introduction to their paper, they suggest that there could be sex differences in vulnerability, stress response, or coping strategies. In their Discussion, they seem to dismiss the possibility that it is merely a result of a sex differential in reporting. They speculate that: a) men may exhibit greater reactivity of the hypothalamic-pituitary-adrenal axis in response to stress exposure than women; b) stressed men may be more reluctant to quit smoking than women; and c) men may be more susceptible to smoking-related diseases. In our analysis, we tested interactions between sex and stress exposure/perceived severity in the fully-adjusted models, but they were not significant. Consistent with Nielsen et al. (2008), the direction of the coefficient suggested that the associations may be stronger for men than women. For example, the implied HR for stress exposure was 1.29 (*p*~0.03) for men versus 1.12 (*p*~0.34) for women, but the sex difference was not significant. We may not have sufficient power to detect sex differences in the association unless they were very large.

Given our relatively small sample of minorities, we have very limited ability to detect interactions with race/ethnicity. In fact, as noted in footnote #1, we were not even able to control for Hispanic ethnicity because MIDUS did not ask respondents to report their ethnicity at Wave 1. Thus, we are not likely to find significant interactions with race because our sample is under-powered (we have only 88 Blacks at Wave 1 and 47 individuals of other race; at Wave 2, we have 243 Blacks, but 72% of them come from the Milwaukee sample and may not be nationally-representative). In any case, we believe it is dangerous to test for interactions without a strong theoretical rationale. If you test enough potential interactions, you will find something that is “significant” merely by chance. Prior work has demonstrated that minorities may be exposed to more stressors, but they did not report higher stress appraisal (Brown, Mitchell, and Ailshire 2020). The authors speculate that minorities may be less likely to appraise a stressor as “stressful” because: a) they have unique psychosocial resources (e.g., social support, religious participation) that provide better coping strategies; b) they have developed more effective coping strategies as a result of earlier and more frequent exposure to

stressors; and/or c) they are actively reframing the stress exposure to lessen its emotional impact.

3. It will be great to include the practice implications of this paper and how it can improve the health and well-being of older adults.

Response: We assume that the reviewer is asking about potential implications for clinical practice. We have added a new sub-section titled “Implications” to the Discussion where we outline the key implications of our results for future research (i.e., importance of controlling for health status as a confounder and evaluating the extent to which the relationship varies by age and cause-of-death) and for clinical practice (i.e., concerns about stress exposure may be better focused on individuals in midlife, subjective questions regarding perceived stress may be susceptible to endogeneity, clinicians could encourage practices that facilitate better coping with stress for those exposed to stressors).

4. Considering the increase in stress levels among younger adults as the study findings indicate, how does this translate to better health outcomes as they age across the lifespan and into older adult years. Are there any implications or recommendations that could positively shape stress and mortality rates among the U.S. population?

Response: It is not clear to us what “study findings” the reviewer is referring to when s/he says, “*Considering the increase in stress levels among young adults as the <u>study findings</u> indicate*…”

Our sample did not target individuals younger than 25, so we cannot say anything about the possible trends among people younger than 25. To determine whether stress levels have increased over time (i.e., a period effect), we would need to compare a same-aged sample across different time periods. Our current analysis is based only on the longitudinal cohort who were aged 26-75 at the 1st wave of the NSDE, aged 35-85 at Wave 2, and 47-93 at Wave 3 (see footnotes to Table 1). One could potentially compare the stress levels in Wave 1 of the NSDE with the corresponding stress levels in the Refresher cohort of a similar age range (25-75) that was interviewed in 2011-14. However, we have not included the Refresher cohort here. Therefore, our results do not allow us to say whether stress levels have increased over time even among adults aged 26-75.

Consequently, we cannot speak to the question the reviewer seems to be raising: have levels of stress among younger people increased over time? If so, what might be the implications for health outcomes among recent cohorts as these younger cohorts grow older?

What our results do show is that stress exposure and perceived stress appear to have a weaker association with mortality at older ages. The implication of this result is that even if younger people who are exposed to stress have higher rates of mortality, the effect of stress exposure may not matter as much as other factors when they reach

older ages. Given that the vast majority of Americans die at ages older than 65 (79.6% based on current period US life tables for 2021; https://www.mortality.org/File/GetDocument/hmd.v6/USA/STATS/bltper_1x1.txt accessed March 21, 2024), this finding seems encouraging.

In our fully-adjusted models, there was virtually no association between exposure to daily stress and mortality by age 70. The same period life tables imply that 72.7% of Americans are expected to die after age 70.

For the ~33% of Americans expected to die below age 70, exposure to stress could be a risk factor. It might may explain why the US has such high levels of midlife mortality, particularly from drug and alcohol use, suicide, and cardiometabolic causes (Harris, Woolf, and Gaskin 2021). If Americans can survive to age 65, they exhibit mortality rates that are similar to (and at ages 85+ are even lower than) other high-income countries (see Barbieri 2019, Figure 2). But in midlife, Americans die at a much faster rate than their counterparts in many other countries. As Barbieri (2019) shows, around age 25, US mortality rates are nearly 2.5 times higher than the average of 18 other high-income countries. Although relatively few Americans die in midlife, those premature deaths have an undue influence on overall life expectancy. Efforts to address factors that may reduce midlife mortality, whether those factors relate to stress exposure or other variables that increase susceptibility to external mortality including the so-called deaths of despair, could benefit US life expectancy and population health.

REVIEWER #2

Thank you for the opportunity to review the manuscript, "Daily exposure to stressors, daily perceived severity of stress, and mortality risk among US adults."

Overall, I thought the authors did a good job explaining the study. The manuscript started with quotes relevant to the study and thought it was a good set up to discuss exposure to stressors and daily perceived severity of stress on mortality risk. I appreciated the supportive files as they help me understand the rationale and reasoning of the authors and study. The tables were excellent. I thought the authors did a great job on constructing the tables with explanations (notes) at the bottom.

I have a few things for the authors to address. In the 2nd paragraph of the intro, "i.e., the extent to which s/he feels..." should include gender neutral language. Can be done by changed the statement to, she/he/they. Same goes for throughout the manuscript.

Response: We have changed “s/he” to “the person.”

In the 3rd paragraph of the intro, "Most studies are based on one-time..." could use citations to let the reader know which studies the authors are referring to.

Response: As requested, we have added citations to that sentence to identify the studies that were based on one-time measurement perceived stress (which comprised

all but two of the studies previously cited). The other two studies (Hange et al. 2013; Vasunilashorn et al. 2013) measured perceived stress at several follow-up waves, but measured it only once at each survey wave. To our knowledge, there is no prior study of perceived stress and mortality that incorporated multiple measurements of perceived stress over a short period. We have added text to the Discussion indicating as such.

In the 2nd paragraph of the measures, I think there might be a typo: analysis should be analytical?

Response: We have changed “analysis sample” to “analytic sample” throughout the manuscript. We believe that “analytical sample” would also be acceptable (although “analytical” seems to be more commonly used as an adjective for a person). If PLOS ONE’s editor (Dr. Bhattacharyya) has a preference, we are willing to change the term accordingly.

In the 4th paragraph of the measures section, perhaps add a citation to be consistent with the previous paragraph.

Response: As noted in the previous paragraph, extrinsic mortality was defined based on Masters et al. (2018) with some modifications. The residual category (i.e., “intrinsic” mortality) encompasses all other deaths; by definition, any death that is not “extrinsic” is “intrinsic.” However, we do not think it would be appropriate to cite Masters et al. (2018) because they never use the term “intrinsic mortality” nor do they even include those deaths in their cause-specific analysis. They focus only on all-cause mortality and on extrinsic mortality.

In the paragraph in question, we have added the clarifications shown in red:

> We refer to the residual category as “intrinsic mortality”, which encompassed all other deaths not defined as “extrinsic.”

We have also added the following sentence:

> Researchers use the term “intrinsic mortality” to refer to deaths believed to result from aging, whereas “extrinsic mortality” denotes deaths thought to stem from environmental hazards, the risk of which are more constant across age (Koopman et al. 2015).

In the Daily exposures to stressors section, the authors did not include ranges, maybe be further elaborate.

Response: It is not clear to us which ranges the reviewer is requesting. The individual questions had a binary response (yes/no). We added a clarification in the text indicating that “(responses were binary: yes/no).” The descriptive statistics (including the observed range) for the final summary measure (per day average number of stressors) are shown in Table 1. We have added that information to the text as well.

In the Daily perceived severity of stressors sections in hard to follow, maybe further elaborate.

Response: It is not obvious to us what elaboration to the description of the daily perceived severity of stress would seem clearer to the reviewer. We realize that the construction of this measure is complicated because we have 7 stressors, each of which were measured daily for up to 8 consecutive days, and we created a summary measure of average severity across days and stressors. We have done the best we can to explain it.

In the discussion, the manuscript would improve if the authors clearly labeled the limitations, but also included the strengths of the study. The authors could expand on the conclusion by including possible implications perhaps.

Response: We have added a label for a new sub-section of the Discussion titled "Limitations." Many of the study's have already been noted in the manuscript, but we added a new sub-section of the Discussion titled "Strengths" just prior to the Limitations sub-section to further highlight them. Finally, we have added a new sub-section titled "Implications" just after the Limitations sub-section (see also our response to Reviewer #1, Point #3).

I am unfamiliar with the guidelines for authors regarding word counts, etc. Some of these comments may not be appliable.

I recommend the manuscript be accepted with minor revisions.

REFERENCES

Barbieri, Magali. 2019. "The Contribution of Drug-Related Deaths to the US Disadvantage in Mortality." *International Journal of Epidemiology* 48(3):945–53. doi: 10.1093/ije/dyy288.

Brown, Lauren L., Uchechi A. Mitchell, and Jennifer A. Ailshire. 2020. "Disentangling the Stress Process: Race/Ethnic Differences in the Exposure and Appraisal of Chronic Stressors Among Older Adults." *The Journals of Gerontology Series B: Psychological Sciences and Social Sciences* 75(3):650–60. doi: 10.1093/geronb/gby072.

Hange, Dominique, Kirsten Mehlig, Lauren Lissner, Xinxin Guo, Calle Bengtsson, Ingmar Skoog, and Cecilia Björkelund. 2013. "Perceived Mental Stress in Women Associated with Psychosomatic Symptoms, but Not Mortality: Observations from the Population Study of Women in Gothenburg, Sweden." *International Journal of General Medicine* 6:307–15. doi: 10.2147/IJGM.S42201.

Harris, Kathleen Mullan, Steven H. Woolf, and Darrell J. Gaskin. 2021. "High and Rising Working-Age Mortality in the US: A Report From the National Academy of Sciences, Engineering, and Medicine." *JAMA* 325(20):2045–46. doi: 10.1001/jama.2021.4073.

Koopman, Jacob J. E., Maarten J. Wensink, Maarten P. Rozing, David van Bodegom, and Rudi G. J. Westendorp. 2015. "Intrinsic and Extrinsic Mortality Reunited." *Experimental Gerontology* 67:48–53. doi: 10.1016/j.exger.2015.04.013.

Nielsen, Naja Rod, Tage S. Kristensen, Peter Schnohr, and Morten Grønbæk. 2008. “Perceived Stress and Cause-Specific Mortality among Men and Women: Results from a Prospective Cohort Study.” *American Journal of Epidemiology* 168(5):481–91. doi: 10.1093/aje/kwn157.

Vasunilashorn, S., D. A. Glei, M. Weinstein, and N. Goldman. 2013. “Perceived Stress and Mortality in a Taiwanese Older Adult Population.” *Stress (Amsterdam, Netherlands)* 16(Journal Article):600–606. doi: 10.3109/10253890.2013.823943.
